# Peltier cell calorimetry “as an option” for commonplace cryostats: Application to the case of MnFe(P,Si,B) magnetocaloric materials

**DOI:** 10.1016/j.fmre.2022.09.020

**Published:** 2022-10-14

**Authors:** J.Y. Xu, F. Guillou, H. Yibole, V. Hardy

**Affiliations:** a College of Physics and Electronic Information, Inner Mongolia Key Laboratory for Physics and Chemistry of Functional Materials, Inner Mongolia Normal University, 81 Zhaowuda Road, Inner Mongolia, Hohhot 010022, China; b Normandie University, Caen 14000, France

**Keywords:** Heat capacity, Calorimetry, Differential scanning calorimeter, Magnetocaloric effect, Magnetocaloric materials, Thermomagnetic materials

## Abstract

Peltier cell calorimetry is a powerful technique to record both the heat capacity and the latent heat, yet its availability is limited as it often requires homemade dedicated cryostats. Here, we describe the development of a Peltier cell differential scanning calorimeter facilitating the accessibility to the technique, since it is designed “as an option” for commonplace commercial cryostats equipped with high magnetic fields. This yields an apparatus well suited to detailed studies of magnetic transitions in general and of first-order magnetic transitions in particular. For magnetocaloric materials, our system can also be used to measure *directly* the isothermal entropy change Δ*S* induced by a magnetic field change; it even allows separating the cyclic (reversible) effect due to successive magnetization/demagnetization, which is the one relevant for applications, from the total magnetocaloric effect. To illustrate the versatility of this system, a thorough study of the ferromagnetic first-order transition of MnFe_0.95_P_0.585_Si_0.34_B_0.075_ is carried out. An exceptionally large cyclic entropy change at an intermediate field is observed in this compound, Δ*S*_cyclic_ = 13.2 J kg^−1^ K^−1^ for µ_0_Δ*H* = 1 T. This confirms that MnFe(P,Si,B) shows one of the most promising giant magnetocaloric effects to be used in emergent green technologies such as magnetocaloric cooling, heating or thermomagnetic waste heat recovery.

## Highlights

• convenient Peltier cell DSC as an option for commercially available cryostats.
• complementarity between magnetization and heat flux data at magnetic transitions.
• direct confirmation of an exceptionally large cyclic entropy change in MnFe(P,Si,B).
• asymmetry of the transformation observed both as a function of T and H.

## Graphical abstract

**Figure:**
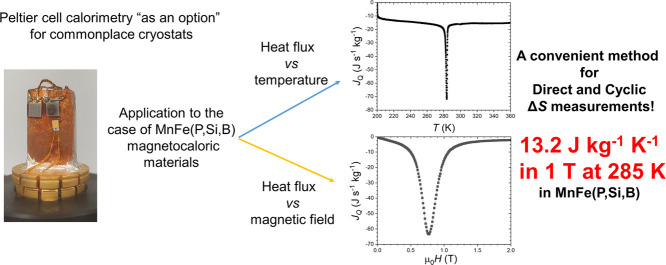

## 1 Introduction

The heat capacity is one of the most basic physical properties and measuring its temperature dependence is of great importance in condensed matter. For studying magnetic materials and their magnetic phase transitions, one needs in addition the possibility to apply an external magnetic field, and to perform calorimetric measurements in high fields, which can be tricky in some systems. In-field heat capacity measurements are especially important for magnetocaloric or thermomagnetic materials since they allow a quantitative estimate of the two main performance parameters: the isothermal entropy change (Δ*S*) and the adiabatic temperature change (Δ*T*_ad_) [1], [2], [3], [4], [5], [6]. On top of that, the heat capacity impacts the amount of heat stored in the material, therefore it is by itself one of the key parameters when dimensioning magnetocaloric refrigerators, magnetocaloric heat pumps or thermomagnetic generators. These latter are currently attracting interest as potential green technologies for cooling, heating or waste heat recovery, respectively [1], [2], [3], [4], [5], [6]. While most prototypes currently operate near room temperature, low temperature applications are also targeted including for gas liquefaction [7], [8], [9], in turn requiring to developing characterization techniques covering a large temperature range.

Experimental measurements of the heat capacity are challenging, in particular near first-order transitions involving a latent heat contribution. Adiabatic, quasi-adiabatic, relaxation or AC calorimeters, consisting in applying a small heat pulse and analyzing the resulting temperature-time response of the material, allow to accurately record the heat capacity. They however encounter serious issues in the presence of latent heat and hysteresis, which requires the use of complementary methods [10], [11], [12], [13], [14]. On the other hand, continuous temperature scans such as that performed in differential thermal analysis or in differential scanning calorimetry (DSC) are well suited to detect first-order transitions and to quantify the associated latent heat. However, common DSCs operate at relatively high sweep rates with an exchange gas (d*T*/d*t* are usually of the order of 10 K min^−1^), which limits their accuracy for determining the heat capacity. In addition, their implementation in a high magnetic field source is not necessarily straightforward [15]. In this context, heat flux calorimetry operating at high vacuum and employing Peltier cells appears as a compromise since it can offer a reasonable accuracy both on latent heat and heat capacity [16], [17], [18], [19], [20], [21], [22], [23], [24], [25], [26]. This type of calorimetry exploits the fact that the heat flux flowing in/out from the sample is proportional to the Seebeck effect generated by the Peltier cell during thermal or magnetic field ramps. It is therefore conceptually different from power compensated DSC in which the difference in applied thermal energy between sample and reference is quantified or usual heat flux DSC in which the heat flux is calculated from the difference between sample and reference temperatures.

The intense efforts paid over the past two decades on the development of magnetocaloric materials and the understanding of their first-order magnetic transitions (FOMT) have led to the development of several Peltier cell calorimeters [16], [17], [18], [19], [20], [21], [22], [23], [24], [25], [26]. However, most of the designs proposed so far either involve the realization of a complete insert for the cryostat with the development of a dedicated temperature control (sometimes including the use of power Peltier cell for temperature control which limits the accessible temperature range) or use electromagnet or permanent magnet as field source which limits the accessible field range. A key aspect of the presently described Peltier cell calorimeter is the simplicity of its conception and use, as it is designed to be operated as an option for the relatively commonplace Quantum Design cryostats such as physical properties measurement systems (PPMS) and Versalab systems. The temperature and magnetic field controls are built-in features of the cryostat (typical temperature range is 2(50)−400 K, while the magnetic field can be as large as 14 T), while the additional external electronics are limited to the measurements of the Seebeck voltage and of the local temperature by means of a resistance temperature detector (RTD). The measurement cell is built on a standard cryostat puck, making the setting up of a new measurement a matter of minutes. Compared to Peltier cell calorimetry previously proposed for Quantum Design systems [24], our calorimeter represents a major evolution on three important aspects: (i) We use two Peltier cells mounted in differential configuration allowing a direct cancelation of most of the baseline signal; (ii) Sample positioning is optimized so as to minimize magnetic torque and demagnetizing effects; (iii) An additional temperature probe allows a better estimate of the sample temperature.

Peltier cell calorimeters are particularly important for research on magnetocaloric materials. They allow to record the heat capacity *C* as a function of temperature *T* in different magnetic fields *H* and to build by integration entropy curves *S*(*T,H*). Their differences at constant temperature or constant entropy provide *indirectly* the isothermal entropy change ΔS(T,ΔH)=S(T,H)−S(T,H=0) and the adiabatic temperature change $\Delta T_{ad}\left(T,\Delta H\right)\;=\;T\left(S,H\right)-T\left(S,H=0\right)$, respectively [1]. In addition, Peltier cell calorimeters can also record the heat flux from/to the sample during a magnetization or demagnetization process at constant temperature. It therefore allows a *direct* measurement of the entropy change resulting from an isothermal (de)magnetization process. Such direct measurements of the entropy change are essential for evaluating the performances of magnetocaloric materials [18], [19], [20], [21], [22], [23], [24], [25], [26], [27], [28], [29], [30], [31]. For instance, they took a determinant role in settling the controversies on the invalidity of the Δ*S* spike. The latter turned out to be an artefact observed around first-order magnetic transitions when calculating the entropy change by applying the Maxwell equation on isothermal magnetization curves. While Δ*S* spikes can be spectacularly large, they reflect the thermal evolution of a ferromagnetic fraction induced by irreversibility which is not a genuine magnetocaloric effect. More recently, direct entropy change measurements also showed that care must be taken in evaluating the entropy change resulting from cyclic magnetization and demagnetization processes. In presence of hysteretic FOMTs –more specifically when considering partial phase transitions– the cyclic (sometimes referred to as reversible) entropy change cannot be straightforwardly estimated from indirect methods, in such a way that direct entropy change measurements are needed [30,31]. While materials deriving from Fe_2_P such as MnFe(P,As), MnFe(P,Ge), MnFe(P,Si) or MnFe(P,Si,B) show amongst the largest magnetocaloric or thermomagnetic effects, their entropy change have so far been evaluated by indirect methods (most often from magnetic data, more scarcely from isofield calorimetric data) [32], [33], [34], [35], [36], [37], [38], [39]. Some direct measurements of the adiabatic temperature change have been reported [35,39,[41], [42], [43]], but hardly any direct entropy change measurements are available for this promising materials family.

The present study seeks to facilitate the access to heat capacity and direct entropy change measurements by describing a simple Peltier cell calorimeter implemented as an option for commercially available cryostats. The capabilities of this calorimeter are illustrated by the case study of a prototypical MnFe(P,Si,B) material with giant magnetocaloric/thermomagnetic effect.

## 2 Experimental details, methods and materials

### 2.1 The Peltier cell calorimeter

**Fig. 1:**
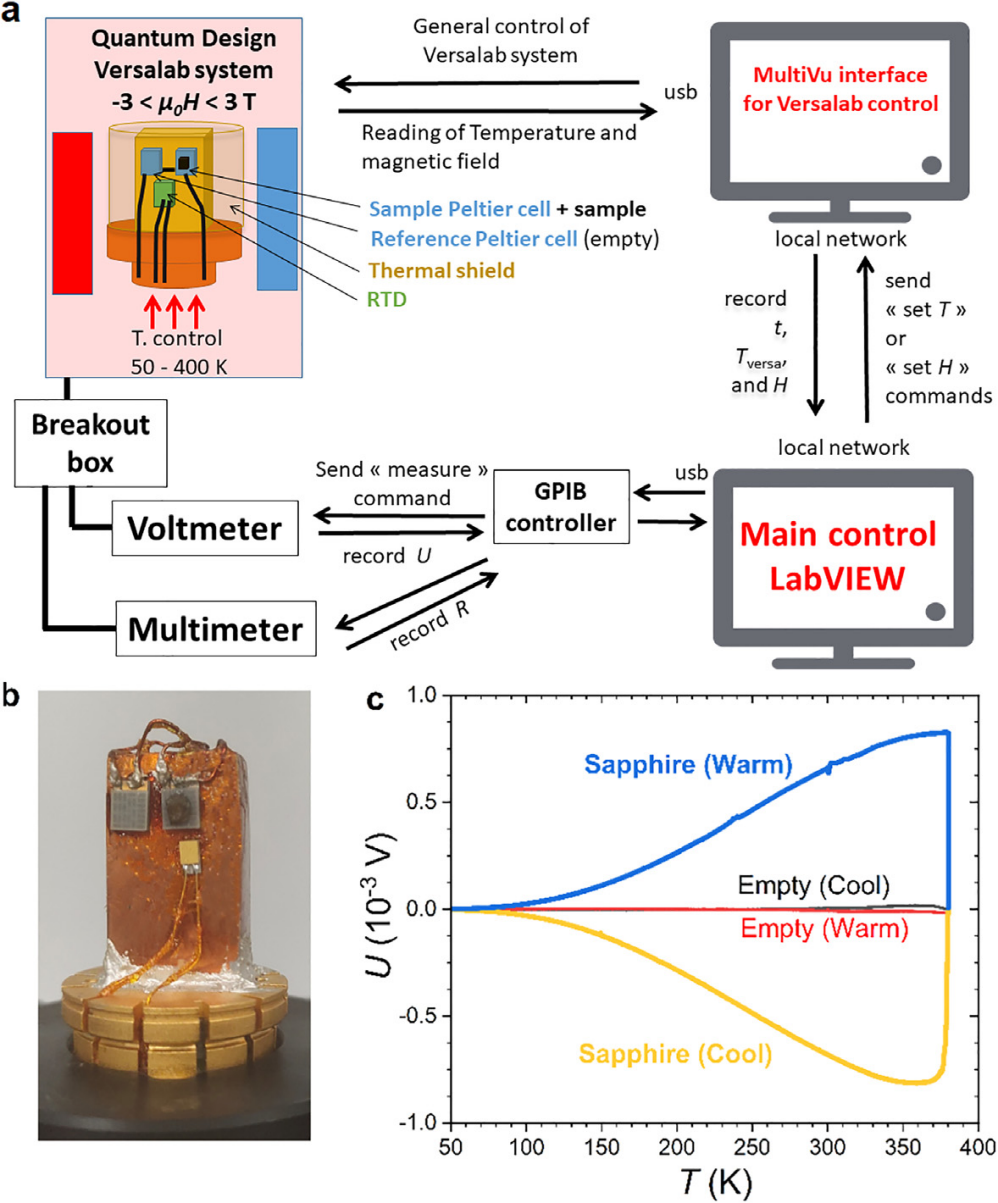
(a) Schematic representation of the Peltier cell calorimeter. (b) Picture of the measurement puck. (c) Raw signals of the calibrating runs at 1.5 K min^−1^ in zero field.

The Peltier cell calorimeter was designed as an option quickly installed/removed for the relatively commonplace Quantum Design cryostats, that can be safely operated by non-expert users. The present study of MnFe_0.95_P_0.585_Si_0.34_B_0.075_ was carried out on a Versalab system, yet a migration to recent Physical Properties Measurements Systems or Dynacool systems does not require any modifications. A schematic block diagram of the overall instrument is shown in Fig. 1. The main control interface is programmed in LabVIEW (2018, National Instruments) running on a separate computer. The functions of the Versalab cryostat (mainly the sample space atmosphere, temperature, and magnetic field) are controlled by the Quantum Design Multivu interface operated in remote mode via LAN network. Keithley Nanovoltmeter Model 2182A and Source Measure Unit Model 2400 connected to the main control interface via GPIB are used for reading the Seebeck voltage and RTD, respectively.

The measurement puck is built using a blank universal sample puck onto which a massive copper block is glued in vertical position with silver epoxy. The function of this copper block is twofold: first, it allows the sample to be at the center of the magnetic coil so as to limit magnetic torque (in a way similar to the vertical sample kit for heat capacity option [40]); Second, it acts as a temperature constant heat sink during isothermal measurements. Two Peltier cells (miniatures thermoelectric elements, RMT, model 1MD03- 024) are mounted using GE varnish and connected in differential configuration. The local temperature is read using a RTD (Cernox CX-1070-SD-HT) pasted with GE varnish in the near vicinity of the sample cell. In operation, a polished copper thermal shield (not shown on the picture Fig. 1b) is anchored to the copper block. Measurements are usually performed at high vacuum (typically 10^−2^ Pa) using the cryopump of the Q.D. cryostat.

The calibration was performed by recording the Seebeck voltage of the empty cells and then that of a 50.0 mg sapphire disk as a function of the temperature using a linear thermal ramp at 1.5 K min^−1^. The raw signal of the empty measurement, corresponding to some extent to a noise level due to an imbalance between the Peltier cell, is ∼1.0 x 10^−5^ V or less. At the same driving rate, the sapphire signal reaches up to 8.6 x 10^−4^ V. Thus a rough estimate of the signal to background ratio is at least of two orders of magnitude.

### 2.2 Data analysis for the Peltier cell DSC and determination of the magnetocaloric effect

Following the seminal work from Saito et al. solving analytically the heat flux equations for differential scanning calorimeters [44], the data analysis for Peltier cell DSC was revisited using different approaches [17,18,[21], [22], [23]]. Here we choose to follow a simplified version of the method proposed by Basso et al. [23]. Owing to its smallness compared to a typical sample signal, the baseline response can be safely neglected (see Fig. 1). The sample heat flux (*J_Q_*) is given by $J_{Q}=K_{cell}\left(U+\tau \frac{dU}{dt}\right)$, where *U* is the measured potential, *t* the time, *K*_cell_ the cell constant determined by calibration, and τ the time constant of the calorimeter accounting for the thermal conductance of the cell. During thermal ramps, the specific heat is then calculated as $C=J_{Q}\left(\frac{dt}{dT}\right)$ with d*T*/d*t* being the temperature (*T*) ramp. The calorimeter time constant *τ* ≈ 10 s was roughly estimated so as to cancel the apparent thermal or magnetic lags where no magnetic or thermal hysteresis is expected (e.g., outside the first-order transition region).

For magnetization data, isothermal entropy changes (Δ*S*) were *indirectly* determined using the common Maxwell equation $\Delta S\left(T,H\right)=\mu_{0}\int_{0}^{H}{\left(\frac{dM}{dT}\right)}_{H^{\prime }}dH^{\prime }$ on different sets of magnetization measurements [1].

For specific heat measurements as a function of the temperature, the isothermal entropy change and the adiabatic temperature change (Δ*T*_ad_) were *indirectly* determined by first building the entropy lines$\;S\left(T,H\right)-S\left(T_{1},H\right)=\int_{T_{1}}^{T}\frac{C(T^{\prime },\;H)}{T^{\prime }}dT^{\prime }$ by integration of the isofield *C*(*T,H*) data starting from a temperature *T_1_* much below the FOMT. Then, the difference between these curves directly reflects the magnetocaloric quantities: Δ*S*(*T*,Δ*H*) *= S(T, H) – S(T, H=0)* and Δ*T*_ad_(*T*,Δ*H*) *= T(S, H)-T(S, H=0)*. In practice, the curves resulting from the *C/T* integration were vertically shifted using the entropy change measured at *T*_1_ from magnetization data ($\Delta S\left(T_{1},H\right)=S\left(T_{1},H\right)-S\left(T_{1},H=0\right))$, since the Maxwell equation can be safely used at such a temperature lying within the reversible regime.

It must be emphasized that DSC heat flux measurements as a function of the magnetic field allow a *direct* measurement of the entropy change by integration of the heat flux $\Delta S\left(T,\Delta H\right)=\frac{1}{T}\int_{0}^{t}J_{Q}\left(t^{\prime }\right)dt^{\prime }$, with *t* = 0 marking the initial magnetic field and *t* the time at which the final magnetic field is reached.

### 2.3 The MnFe(P,Si,B) sample used as case study

**Fig. 2:**
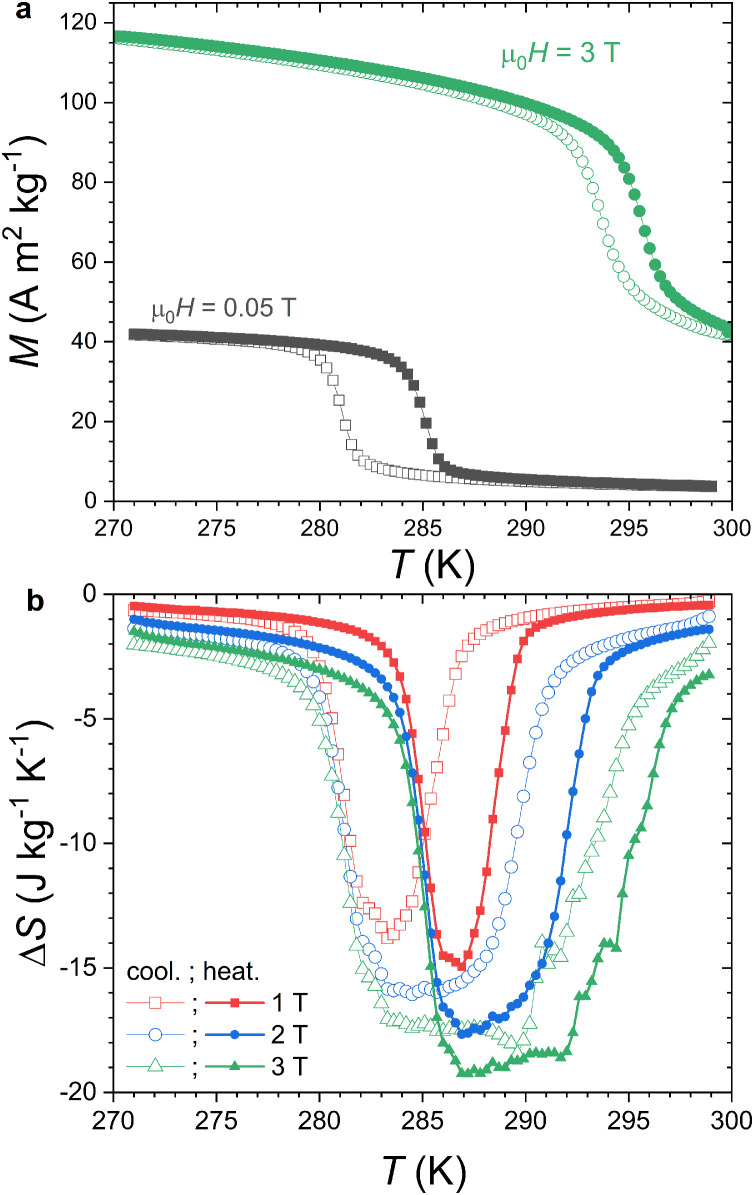
**Isofield magnetic characterization of MnFe_0.95_P_0.585_Si_0.34_B_0.075_.** (a) *M*_H_(*T*) curves recorded upon cooling (open symbols) and heating (closed symbols) at 1 K min^−1^ (no thermal lag correction) in μ_0_*H* = 0.05 and 3 T. (b) Isothermal entropy change Δ*S*(*T*) calculated from a set of *M*_H_(*T*) curves recorded at the same rate with a field increment of μ_0_δ*H* = 0.25 T between 0.25 and 3 T.

A piece (∼1.8 x 1.8 x 1.0 mm^3^, 16.0 mg) of MnFe_0.95_P_0.585_Si_0.34_B_0.075_ representative for one of the most promising magnetocaloric effect achievable at intermediate magnetic field (1–2 T) in the Fe_2_P-related materials family was used to test the Peltier cell calorimeter [35]. This sample batch was prepared by ball-milling followed by solid state reaction at high temperature, ending by a quenching in room temperature water. We note that while this synthesis method is common to most other MnFe(P,Si,B) studies, each sample batch shows minor deviations and is therefore unique. The present sample was taken from the same batch as ref. [45]. The refinement of the powder x-ray diffractogram revealed the presence of a minor secondary phase resembling a Fe_3_Si alloy whose content is estimated at about 3.5 wt%. Since this minor secondary phase is recurrent in MnFe(P,Si) or MnFe(P,Si,B) materials and its content is limited, its influence was disregarded. MnFe_0.95_P_0.585_Si_0.34_B_0.075_ presents a first-order ferromagnetic transition near room temperature with a transition temperature at *T*_C_ ≈ 285 K and a thermal hysteresis of approximately 2 K (see Fig. 2), in reasonable agreement with the properties observed in closely related compositions [35].

Isothermal entropy changes (Δ*S*) were indirectly determined from various sets of magnetization data. First, isofield magnetization measurements *M*_H_(*T*) measured upon heating and cooling at a sweeping rate of +/- 1 K min^−1^ were employed to derive the isothermal entropy change shown in Fig. 2b. Δ*S* from isofield magnetization data are known not to suffer from the spike artefact around FOMT [46]. The resulting Δ*S*(*T*) curves show a relatively standard tower shape with a thermal hysteresis between heating and cooling series. The maximal Δ*S* in μ_0_*H* = 1 T are 15.0 (14.0) J kg^−1^ K^−1^ upon heating(cooling) and the full with at half maximum (*FWHM*) about 3.6 (4.5) K.

**Fig. 3:**
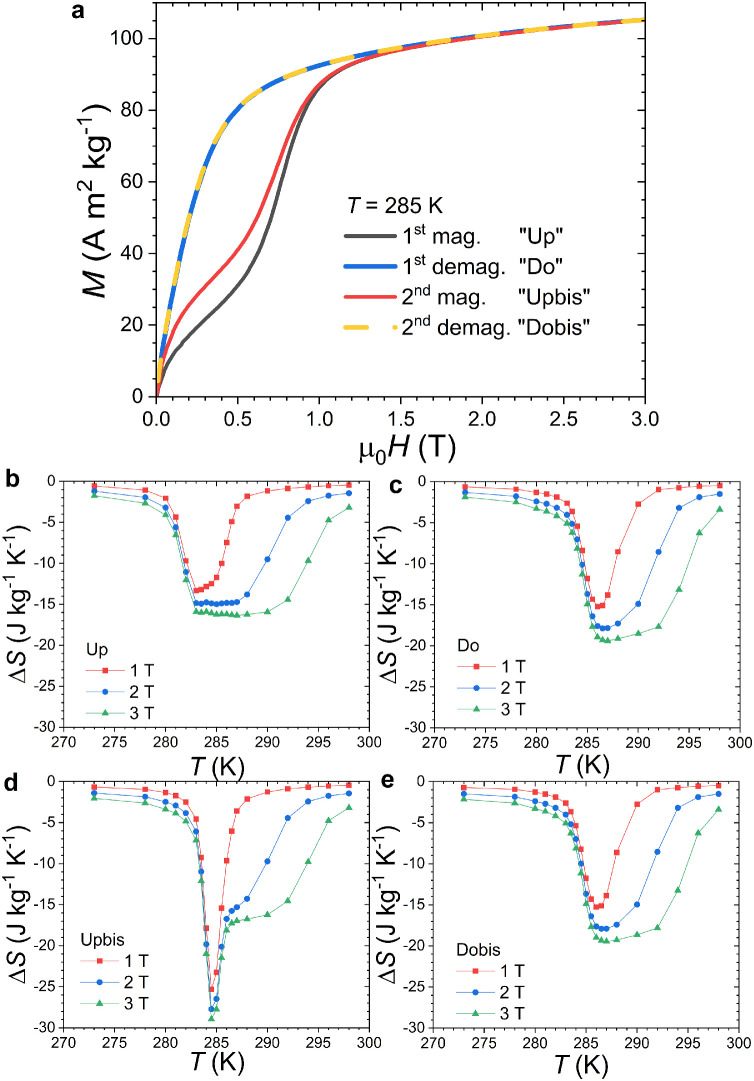
(a) Isothermal magnetization curves *M_T_*(*H*) for MnFe_0.95_P_0.585_Si_0.34_B_0.075_. At each temperatures 4 magnetic curves were recorded after a zero field cooling leading to 4 isothermal entropy changes Δ*S*(*T*) panels: (b) first magnetization (0 → 3 T, Up), (c) first demagnetization (3 T → 0, Do), (d) second magnetization (0 → 3 T, Upbis), and (e) second demagnetization (3 T → 0, Dobis).

A set of magnetization *M*_T_(*H*) measurements was carried out at constant temperature along 4 branches within the same quadrant after a zero field cooling (see Fig. 3): upon magnetizing (0 → 3 T, Up), demagnetizing (3 T → 0, Do), re-magnetizing (0 → 3 T, Upbis) and a second demagnetizing (3 T → 0, Dobis). The resulting Δ*S*(*T*) curves turn out to be much different between magnetization, demagnetization and second magnetization branches. Only demagnetization and second demagnetization data are reproducible, while the second magnetization (Upbis) obviously suffers from the spike artefact. The later originates from the thermal evolution of a ferromagnetic fraction induced by irreversibility and is not a genuine magnetocaloric effect. The various parameters of the Δ*S*(*T*) curves indirectly derived from magnetization measurements are summarized in Table 1. In general, as expected from the usual hysteresis diagram around a ferromagnetic transition, isothermal magnetization data are comparable to the isofield cooling curves and demagnetization *M*_T_(*H*) branch comparable to heating *M*_H_(*T*) curve.

**Table 1:** **Isothermal entropy change of MnFe**_**0.95**_**P**_**0.585**_**Si**_**0.34**_**B**_**0.075**_**determined indirectly from different sets of magnetization or semi-adiabatic heat capacity data: maximum entropy change (Δ*****S***_**max**_**), full width half maximum of the Δ*****S*****(*****T*****) curve (*****FWHM*****) and the middle of the Δ*****S*****peak (*****T***_**peak**_**).**

Data	Δ*S*_max_	*FWHM*	*T*_peak_	Δ*S*_max_	*FWHM*	*T_peak_*
	J kg^−1^ K^−1^	K	K	J kg^−1^ K^−1^	K	K
		
	Δµ_0_*H* = 1 T			Δµ_0_*H* = 2 T		
*M*_H_(*T*) Heat.	15.0	3.6	286.5	17.7	7.1	289.1
*M*_H_(*T*) Cool.	14.0	4.5	283.1	16.1	9.2	285.4
*M*_T_(*H*) Up	13.4	4.6	284.0	15.0	9.6	285.1
*M*_T_(*H*) Do	15.3	3.9	286.5	18.0	7.6	288.5
*M*_T_(*H*) Upbis	25.3	2.2	285.0	27.8	4.5	286.5
*M*_T_(*H*) Dobis	15.3	3.9	286.5	18.0	7.5	288.5
H.C. “2 τ”	8.2	4.8	286			
H.C. SPM	14.9	4.0	285.7			

**Fig. 4:**
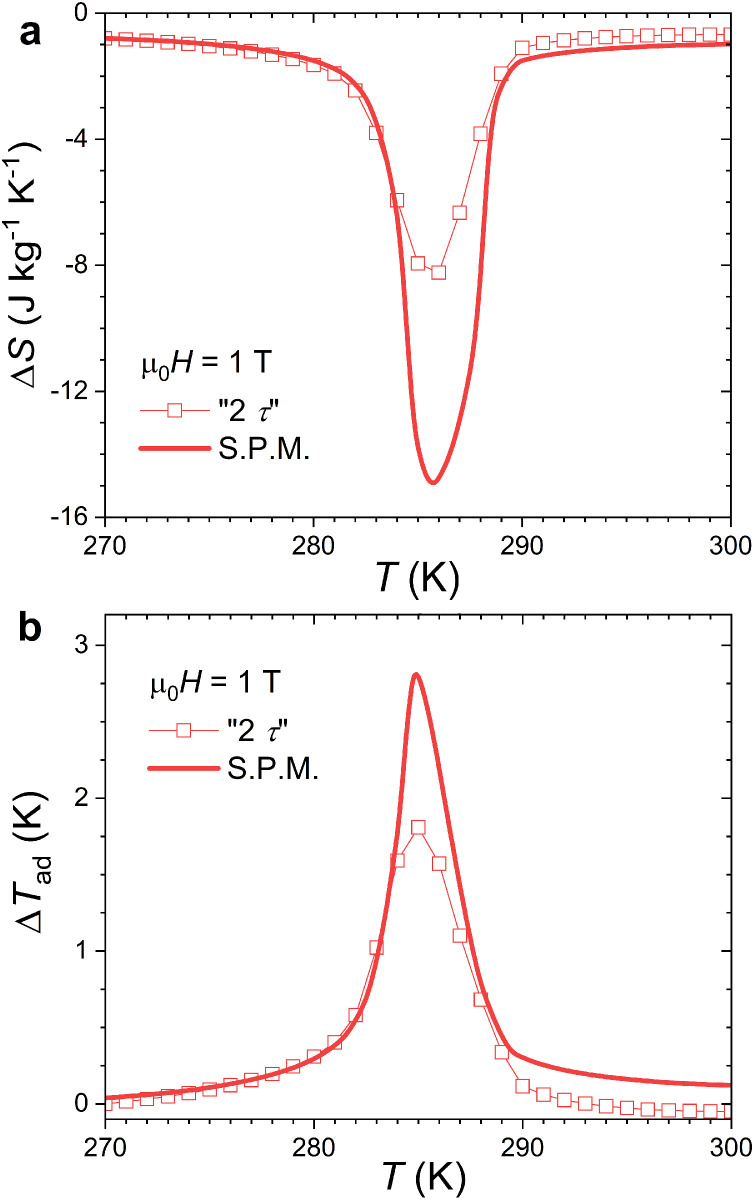
(a) Isothermal entropy change indirectly determined from semi-adiabatic heat capacity data recorded in μ_0_*H* =0 and 1 T using the standard “2τ” method from the Quantum Design heat capacity option or using an external analysis (Single Pulse Method). (b) Adiabatic temperature change indirectly determined from the same dataset as panel (a).

Heat capacity measurements were carried out as a function of the temperature in μ_0_*H =* 0 and 1 T using the semi-adiabatic heat capacity option built-in the Quantum Design cryostat. Fig. 4 presents the Δ*S* and Δ*T*_ad_ indirectly determined from these *C*(*T*). Two sets of data are considered, the standard “2 τ” method provided by the heat capacity option or an external point by point analysis in the transition region referred to as Single Pulse Method (SPM) [12]. The standard “2 τ” method is known to underestimate the latent heat in case of FOMT [10,12,13], which leads to a significantly underestimated magnetocaloric effect. The SPM analysis allows a better estimate of the latent heat leading to a Δ*S* maximum comparable to that obtained from magnetic data, see Table 1. The maximum Δ*T*_ad_ is about 2.8 K for μ_0_Δ*H* = 1 T.

## 3 Results and discussion

### 3.1 In-field DSC measurements as a function of temperature

**Fig. 5:**
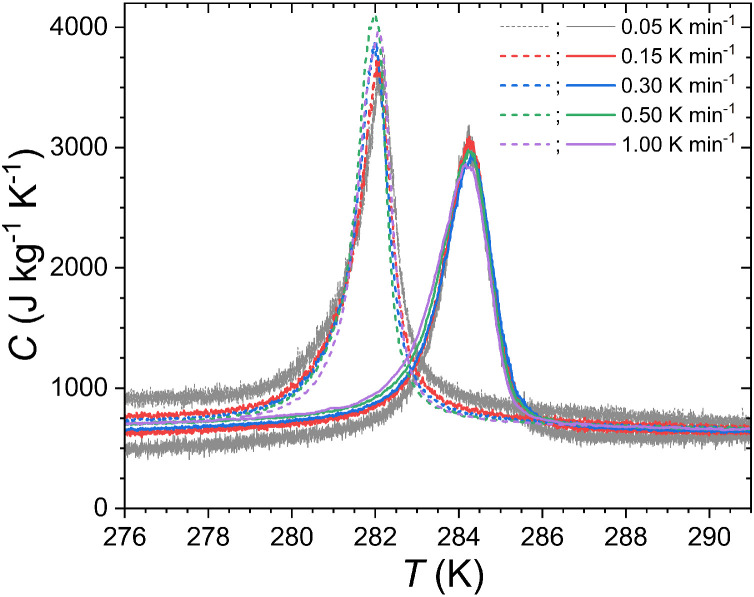
**Specific heat of MnFe**_**0.95**_**P**_**0.585**_**Si**_**0.34**_**B**_**0.075**_**, in zero magnetic field (*****H*****= 0) measured at various temperature rates (d*****T*****/d*****t*****) upon heating (full lines) and cooling (dashed lines).**

The ability of Peltier cell calorimeters to accurately measure the heat capacity as a function of the temperature requires a compromise on the thermal ramp. On the one hand, the thermal ramp should be large enough so that the losses (mainly radiative) remain negligible compared to the heat flux through the Peltier cell; while on the other hand, the rate of the thermal ramp must remain moderate since the thermal lag increases with it. Fig. 5 illustrates the measurement of the heat capacity as a function of the temperature for different thermal ramps around the ferromagnetic transition of MnFe_0.95_P_0.585_Si_0.34_B_0.075_. First, considering the latent heat peak at the FOMT, the peak is well determined and the thermal hysteresis can be well accounted for by measurements upon heating and cooling. The observed thermal hysteresis of 2.1 K is comparable with that measured by other techniques [45]. The possibility to measure hysteretic latent heat peak is an advantage of scanning DSC techniques over heat pulse calorimetry methods. Then, after thermal lags corrections are applied, the transition temperatures and peak shapes remain consistent between measurements at different scanning rates. We however note that measurements at 2 K min^−1^ or faster lead to large corrections and are therefore best avoided. Now when considering the specific heat outside the FOMT, slow thermal ramps such as 0.05 and 0.15 K min^−1^ lead to the appearance of an unphysical opening between heating and cooling branches, indicating that one reaches the low limit for the thermal ramp. At thermal ramps in the range 0.5–1.5 K min^—1^, the heating and cooling branches well overlap outside the FOMT. The deviation between the specific heat measured by DSC (0.5 K min^−1^) and that measured from heat pulse semi-adiabatic calorimetry (in principle more appropriate for heat capacity measurements outside the FOMT with a typical accuracy better than 2%) is between 2% and 6% over the investigated temperature range (200 to 360 K) [45]. Beyond the widely recognized ability of DSC techniques to account for the latent heat, these results show that our Peltier cell DSC can also yield relatively accurate values of the specific heat outside the transitional regime.

**Fig. 6:**
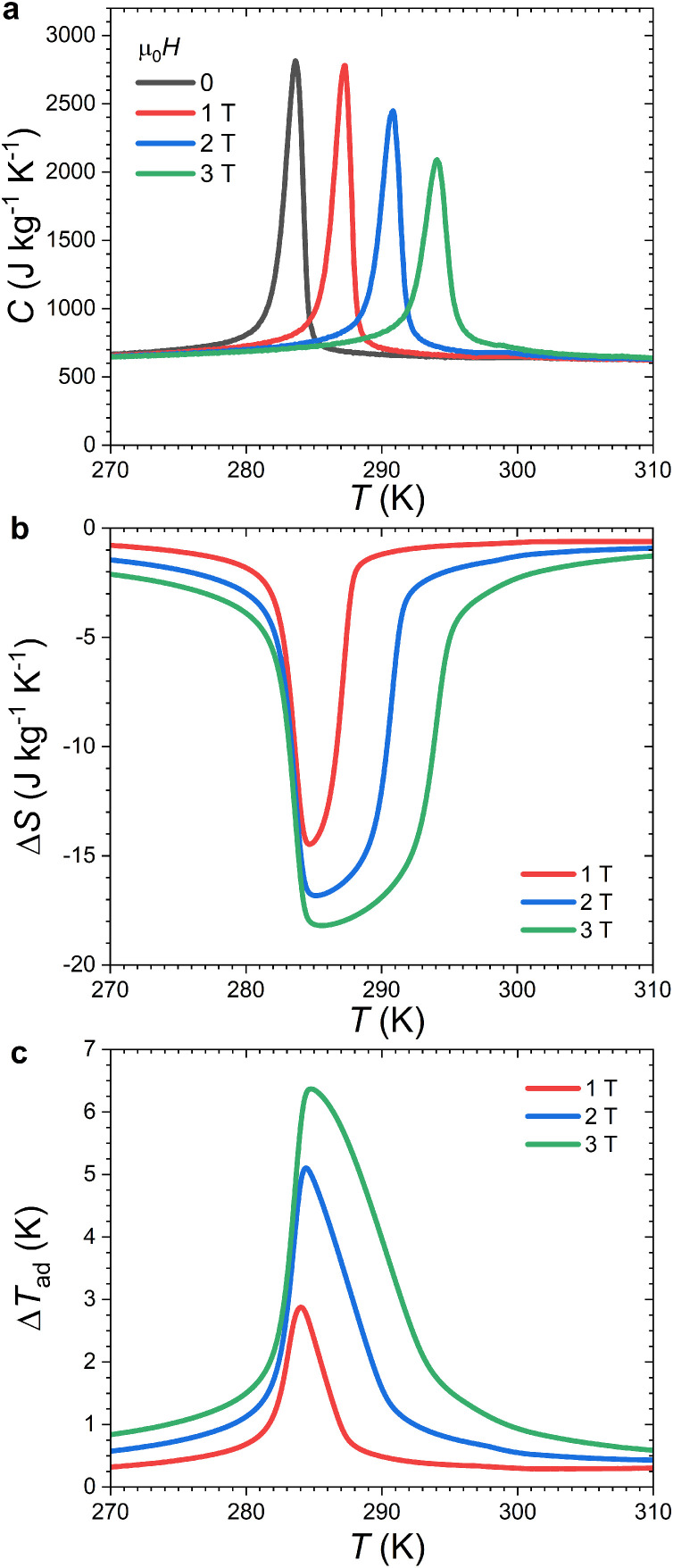
**Magnetocaloric effect of MnFe_0.95_P_0.585_Si_0.34_B_0.075_ indirectly determined from *C*(*T,H*) Peltier cell DSC data.** (a) Specific heat measured in various magnetic fields upon heating (d*T*/d*t* = +1.5 Kmin^−1^). (b) Isothermal entropy change upon heating. (**c**) Adiabatic temperature change upon heating.

Specific heat measurements in applied magnetic fields can be used to indirectly determine the magnetocaloric Δ*S* and Δ*T*_ad_ quantities. This possibility is illustrated for MnFe_0.95_P_0.585_Si_0.34_B_0.075_ in Fig. 6. A good reproducibility of the specific heat background outside the FOMT is an important prerequisite for reliable Δ*S* and Δ*T*_ad_ data. This target is reached with an almost overlap of the *C*(*T*) curves far enough from the transition where the field dependence of *C* is expected to vanish. At the FOMT itself, the shift (d*T*_tr_/μ_0_d*H* = +3.6 K T^−1^) and a progressive broadening of the latent heat peak as the field is increased are typical features of a ferromagnetic FOMT. After integration of the entropy *S*(*T*) lines and their difference at constant temperature (or entropy), the resulting isothermal entropy change Δ*S* reaches 14.5 (15.4) J kg^−1^ K^−1^ for μ_0_Δ*H* = 1 T upon heating (cooling), which are in reasonable agreement with the Δ*S* indirectly measured from magnetization or semi-adiabatic heat capacity data (see Table 1). The adiabatic temperature change Δ*T*_ad_ reaches a maximum of 2.9 K for μ_0_Δ*H* = 1 T, a value close to that estimated using the semi-adiabatic heat capacity option in Section 2.3 or measured by direct methods on comparable compositions [35,42].

### 3.2 Heat flux measurements as a function of the magnetic field

**Fig. 7:**
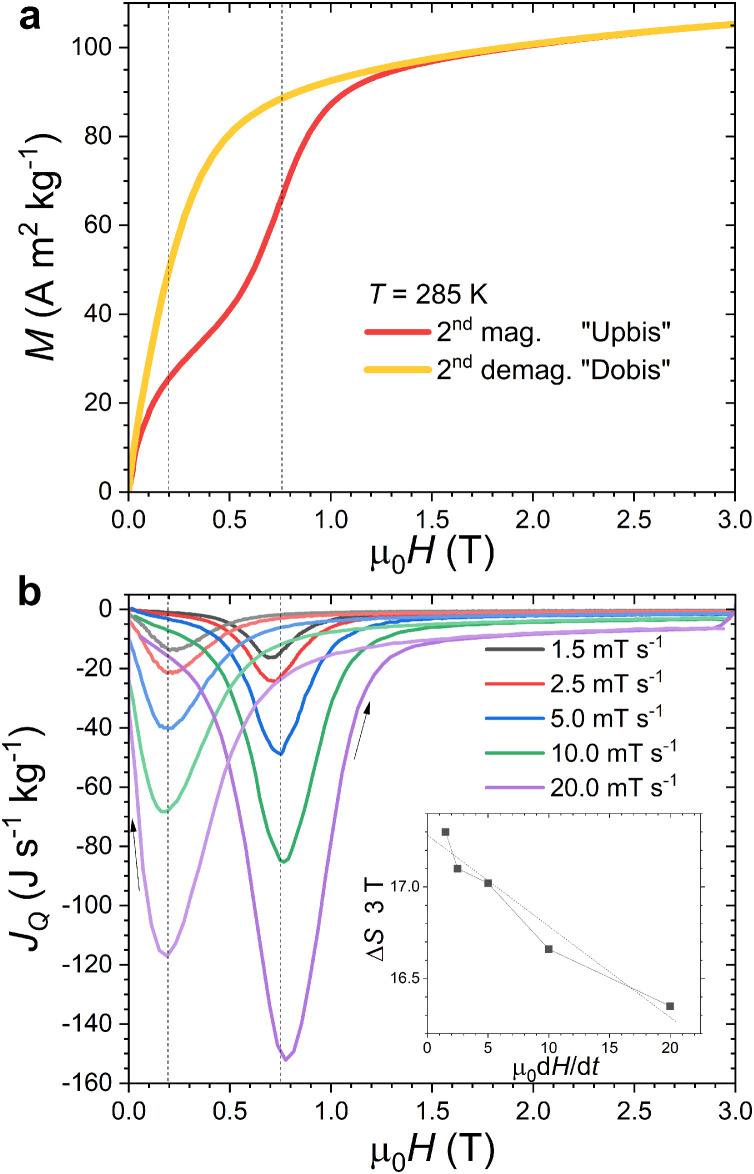
(a) Isothermal magnetization measurements *M*_T_(*H*) across the FOMT of MnFe_0.95_P_0.585_Si_0.34_B_0.075_ at μ_0_d*H*/d*t =* +/- 10.0 mT s^−1^. The vertical dashed lines indicate the center of the FOMT at μ_0_*H* ≈ 0.75 T upon magnetizing and μ_0_*H* ≈ 0.20 T upon demagnetizing. (b) Isothermal heat flux measurements (second magnetization Upbis, second demagnetization Dobis with a sign change) at various μ_0_d*H*/d*t* rates. In inset, the isothermal entropy change at 3 T and 285 K ($\Delta S_{T}\;\left(3T\right)=\frac{1}{T(dH/dt)}\int_{0}^{3T}j_{Q}\left(H\right)dH$) is presented as a function of the magnetic field rate.

One nearly unique capability of Peltier cell calorimeters is to measure the heat flux during an isothermal magnetization/demagnetization, allowing a *direct* determination of the magnetocaloric entropy change. Fig. 7 illustrates the complementarity between isothermal magnetization and heat flux data at the FOMT of MnFe_0.95_P_0.585_Si_0.34_B_0.075_. *M*_T_(*H*) data mostly reflect the phase fraction undergoing the FOMT, for instance, upon magnetization the field-induced paramagnetic to ferromagnetic transition corresponds to an increase in magnetization proportional to the fraction of ferromagnetic phase being formed. On the other hand, the heat flux calorimeter monitors the exchange of heat associated with the phase transformation, but it does not reflect the transformed phase fractions. Upon magnetizing at 285 K, the maximum heat flux is reached around μ_0_*H* = 0.75 T which corresponds to the middle of the magnetization jump on *M*_T_(*H*) data. The operation of the Peltier cell calorimeter in a superconducting magnet offers access to a relatively broad range of field rates, from about one tenth of mT s^−1^ up to approximately 30 mT s^−1^. Yet, similarly to isofield measurements, isothermal heat flux measurements involve a compromise on the magnetic field rate. It must be large enough to generate a measurable tension while remaining reasonably slow to ensure quasi-isothermal conditions. The dependence of Δ*S* on the magnetic field rate is illustrated in the inset of Fig. 7. Fast field ramps lead to a reduction of the estimated Δ*S*, but the deviation compared to the extrapolation toward static conditions remains less than 2% for the ramps at 5 mT s^−1^ or slower. The deviation from isothermal conditions may also be estimated from the temperature difference between the sample temperature and *T*_out_ by the Seebeck effect. For 5 mT s^-1^ at 285 K, the peak tension at the FOMT, *U*_max_ = 4.8 10^−4^ V would correspond to a temperature difference of approximately 0.06 K (approx. 0.08 K when accounting for the balance between the specific heats of the sample and the top plate of the Peltier element), i.e. about two percent or less of the adiabatic temperature change, confirming that nearly isothermal conditions are ensured at 5 mT s^−1^ or slower.

**Fig. 8:**
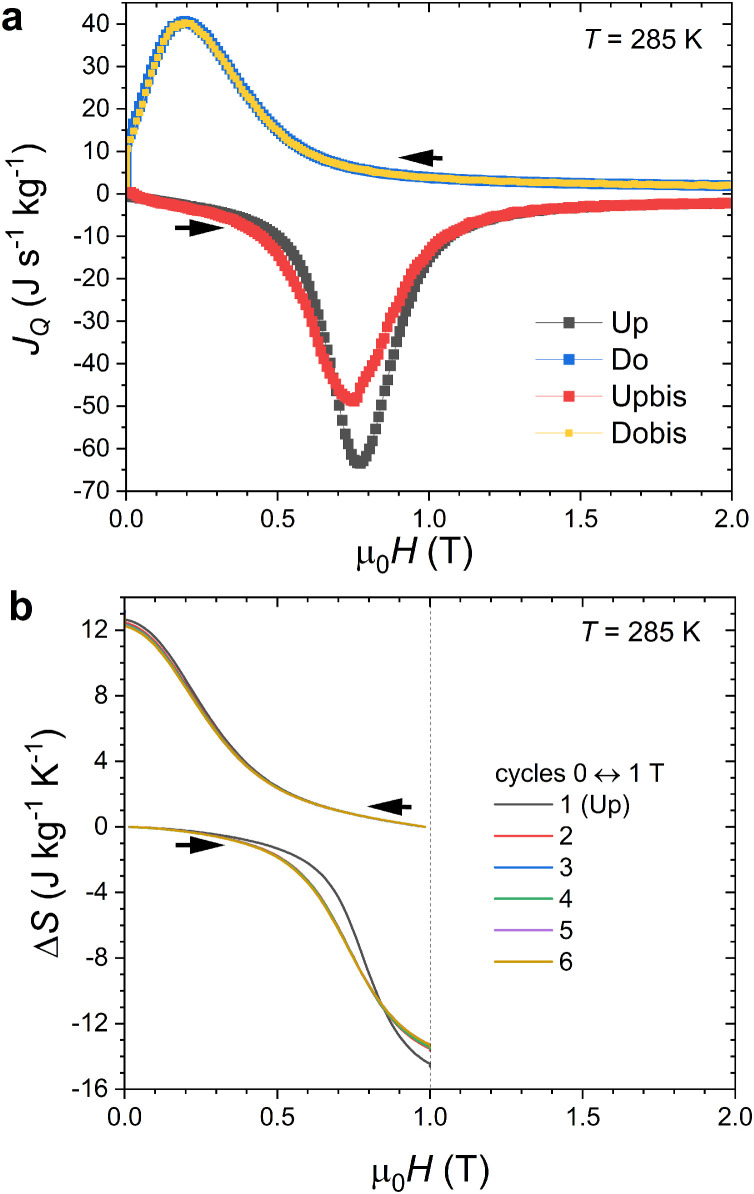
(a) Isothermal heat flux measurements across the FOMT of MnFe_0.95_P_0.585_Si_0.34_B_0.075_ at 285 K (μ_0_d*H*/d*t =* +/- 5.0 mT s^−1^). (b) Direct and cyclic isothermal entropy changes $\Delta S\left(T,\Delta H\right)=\frac{1}{T(dH/dt)}\int_{0}^{1\;T}j_{Q}\left(H^{\prime }\right)dH^{\prime }$ from heat flux measurements 0 ↔ µ_0_*H*_max_ = 1 T (μ_0_d*H*/d*t =* +/- 5.0 mT s^−1^). Note that the curves for cycles 2 to 6 almost perfectly overlap.

On heat flux versus field curves shown in Figs. 7b and 8, we can notice that the magnetizing branches corresponding to the paramagnetic to ferromagnetic transition appear to be sharper than the demagnetizing ones. Similarly, the cooling *C*(*T*) curves on Fig. 5 show a sharper profile than the heating ones. The fact is that sharper transitions upon cooling than upon heating can actually be observed in several DSC or calorimetric studies of MnFe(P,Si,B) materials [35,41,45]. The present isothermal heat flux data confirms such an asymmetry of the FOMT. This feature may originate from the influence of stresses on the nucleation/growth process at the FOMT and/or from the role that magnetic interactions can play in the process of the transformation as observed in La(Fe,Si)_13_ materials [47].

Direct heat flux and isothermal entropy change measurements allow investigating the reversibility of the magnetocaloric effect upon successive magnetization/demagnetization cycles. Irreversibility manifests itself mostly in two aspects; First, there can be an opening between the magnetization and demagnetization branches which reflects a shift in the field value required to trigger the transition; Second, the sample may not fully retransform to its original state after demagnetization. In this latter case, the magnetization will be higher on the second magnetization *M*_T_(*H*) branch than on the first magnetization curve, see Fig. 7a. This partial irreversibility of the FOMT leads to a smaller phase fraction being transformed at the second magnetization cycle, and therefore results in a smaller heat flux. Panel (a) of Fig. 8 illustrates the case of a field change of 2 T at 285 K, with the heat flux or its integrated entropy change being smaller at demagnetisation (Δ*S*_Do_(2 T) ≈ Δ*S*_Dobis_(2 T) ≈ 15.8 J kg^-1^ K^−1^) or re-magnetizsation (Δ*S*_Upbis_(2 T) ≈ −15.9 J kg^−1^ K^−1^) than at the first magnetization after a zero field cooling (Δ*S*_Up_(2 T) ≈ −17.0 J kg^−1^ K^−1^). Assessing the irreversibility of the magnetocaloric effect is particularly important at intermediate magnetic field such as 1 T because: *i)* it is the magnetic field range targeted for magnetocaloric refrigeration or thermomagnetic energy harvesting applications and *ii)* it corresponds to only partial transformations (minor loops) where the cyclic (reversible) magnetocaloric effect usable in applications cannot be estimated from indirect methods [30,42]. Panel (b) of Fig. 8 illustrates cyclic heat flux measurements for MnFe_0.95_P_0.585_Si_0.34_B_0.075_ between 0 ↔ 1 T (6 magnetization/demagnetization cycles are presented). Even if irreversibility affects the entropy change after the first cycle, the decrease remains limited. A large cyclic entropy change of 13.2 J kg^−1^ K^−1^ is still observed for μ_0_Δ*H* = 1 T at *T* = 285 K.

**Fig. 9:**
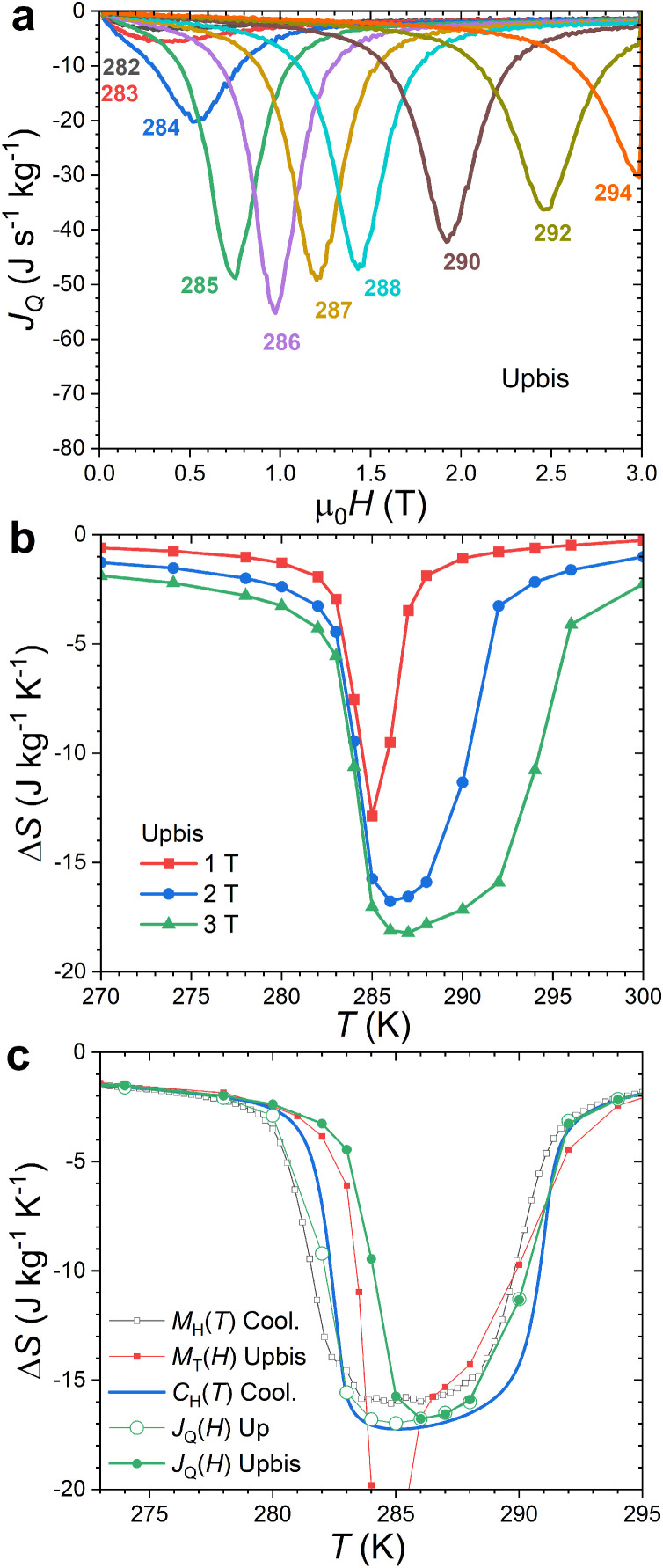
**Isothermal entropy changes measurements of MnFe_0.95_P_0.585_Si_0.34_B_0.075_.** (a) Second magnetization heat flux (Upbis) and (b) their corresponding *direct* cyclic entropy changes. (c) Comparison of the isothermal entropy change Δ*S*(2 T) from various methods for para. to ferromagnetic transition (upon magnetizing or cooling).

Direct isothermal entropy change measurements were repeated at various temperatures for MnFe_0.95_P_0.585_Si_0.34_B_0.075_. Panels a and b of Fig. 9 illustrate the second magnetization data (Upbis), more relevant for the cyclic applications targeted for magnetocaloric materials. The heat flux data reflects the progressive broadening of the FOMT when increasing the applied field. They also reveal that the latent heat peak can be induced by magnetic field as low as 2 T, and most of the peak is actually induced by a magnetic field of 1 T, which is the primary reason for the particularly large entropy change observed at intermediate fields in MnFe_0.95_P_0.585_Si_0.34_B_0.075_. Isothermal entropy changes from various methods are compared in panel c of Fig. 9. While indirect entropy change measurements from *M*_H_(*T*) or *C*_H_(*T*) data are representative for the total effect, only the direct entropy change measurements from *J_Q_*(*H*) data are able to quantify the effect of irreversibility between the first magnetization and subsequent cycles. Magnetic *M*_T_(*H*) Upbis data have a cyclic thermomagnetic history, but they cannot yield the isothermal entropy change due to the presence of the spike artefact. In the present sample, irreversibility only moderately affects the maximal entropy change (a 10% reduction of Δ*S*_max_ is observed for 1 T), it has however a significant impact on the temperature range where a sizable Δ*S* can be observed. The width of the Δ*S*(*T*) peak is reduced between Up and Upbis data by about 2 K, a value comparable to that of the thermal hysteresis in zero field.

The cyclic entropy changes of MnFe_0.95_P_0.585_Si_0.34_B_0.075_ at intermediate fields (1 and 2 T) are compared with similar data from the literature in Table 2. We acknowledge that this comparison is not exhaustive due to the scarcity of direct cyclic Δ*S* data in the literature. We may nonetheless draw some general tendencies. Materials with second-order transitions, such as Gd metal which is the prototypical magnetocaloric material, show fully reversible Δ*S* spread over a large temperature window but exhibit limited Δ*S* maximum. First-order magnetic transitions usually reach large Δ*S* maximum, but often at the expense of a large hysteresis, which in turn limits the exploitable Δ*S*. This is for instance the case for Gd_5_Si_2_Ge_2_ or Heusler alloys requiring magnetic field of 2 T or more to show sizable cyclic Δ*S*. At the end, the particularly large cyclic Δ*S* of MnFe_0.95_P_0.585_Si_0.34_B_0.075_ at intermediate field, 13.2 J kg^−1^ K^−1^ for 1 T, is only equated by La(Fe,Si)_13_H_x_ or FeRh alloys. The present direct entropy change measurements therefore confirm the high interest of MnFe(P,Si,B) compounds for magnetocaloric or thermomagnetic applications. The large Δ*S* values compared to previous studies on MnFe(P,Si,B) originate from the sharpness of the FOMT observed in the present sample batch [35,42]. Despite a sharp transition, MnFe_0.95_P_0.585_Si_0.34_B_0.075_ still shows a full width at half maximum of the cyclic Δ*S* peak of 3.1 K, which remains comparable to the 3 K-wide typical temperature span usually considered as operating window for one single layer of materials for magnetic refrigeration [[1], [2], [3], [4], [5], [6],[48], [49], [50]]. Covering a larger temperature span in applications would however require a gradient of transition temperatures and therefore of compositions.

**Table 2:** **Cyclic (reversible) isothermal entropy change of MnFe**_**0.95**_**P**_**0.585**_**Si**_**0.34**_**B**_**0.075**_**determined from direct isothermal heat flux measurements and comparison with other promising magnetocaloric materials families: Maximum entropy change (Δ*****S***_**max**_**), full width half maximum of the Δ*****S*****(*****T*****) curve (*****FWHM*****), and peak temperature (*****T***_**peak**_**).**

Material	Δ*S*_max_	FWHM	*T*_peak_	ΔS_max_	FWHM	*T*_peak_	Ref.
units	J kg^−1^ K^−1^	K	K	J kg^−1^ K^−1^	K	K	
			
	Δµ_0_*H* = 1 T			Δµ_0_*H* = 2 T			
MnFe_0.95_P_0.585_Si_0.34_B_0.075_	13.2	3.1	285	16.6	7.0	286	Present
Gd	2.9	35(0.9 T)	293	4.0(1.5 T)	45(1.5 T)	293	[21]
Gd	2.8	35	294	–	–	–	[26]
Gd_5_Si_2_Ge_2_	1.8	6	272	8	10	272	[24]
Gd_5_Si_2_Ge_2_	–	–	–	11(1.5 T)	7(1.5 T)	274	[26]
Gd_5_Si_2.09_Ge_1.91_	3.5	–	288	9.0	–	288	[21]
Gd_5_Si_2_Ge_1.9_Ga_0.1_	2.2	28	295	4.0	41	295	[28]
La_1_(Fe−Co−Si)_13_	4.6	14	293	–	–	–	[23]
LaFe_11.05_Co_0.91_Si_1.04_	1.8 b	–	282	10.5 b		282	[48]
LaFe_11.74_Co_0.13_Si_1.13_	–	–	196	20.3 b	–	196	[48]
LaFe_11.84_Mn_0.34_Si_1.30_H_x_	10.6 b	–	292	13.0 b	–	292	[48]
LaFe_11.83_Mn_0.32_Si_1.30_H_x_	11.1 b	–	297	12.6 b	–	297	[48]
Fe_49_Rh_51_	9.4 b		317	13.2 b		317	[48]
Ni_50_Mn_36_Co_1_Sn_13_^a^	–	–	–	1.7(1.5 T)	–	320	[23]
Ni_41.5_Co_9.2_Mn_32_Ga_14_In_3.3_	–	–	–	11 (6 T)	17	335	[25]
Ni_42.47_Co_8.87_Mn_31.67_Ga_14.98_In_2.01_	–	–	–	8 (6 T)	6	410	[25]
Ni_45.7_Mn_36.6_In_13.5_Co_4.2_	–	–	–	10.5	–	278	[30]
(MnNiSi)_0.56_(FeNiGe)_0.44_	4.7	7.2	285				[31]

a values given for *T*_C_ of the austenite phase, not at the martensitic transition showing nearly negligible cyclic Δ*S*.

b indirectly estimated from isofield measurements, not from direct cyclic entropy change data.

## 4 Conclusion

A Peltier cell calorimeter to be used as an option for commonplace commercial cryostats is presented. Peltier cell calorimetry is a convenient approach to record both the heat capacity and the latent heat as a function of the temperature in an applied magnetic field, allowing detailed studies of magnetic transitions in general and of first-order magnetic transitions in particular. For magnetocaloric materials, the reported Peltier cell calorimeter facilitates performing direct entropy change measurements and allows determining the cyclic (reversible) entropy change associated with successive magnetization/demagnetization processes. Peltier cell calorimetry is complementary to magnetization measurements. The former provides information on the heat exchange during the magnetic field change, while the latter reflects the phase fraction undergoing the transformation.

The performances of the calorimeter are illustrated through a detailed study of the ferromagnetic first-order transition of a prototypical MnFe_0.95_P_0.585_Si_0.34_B_0.075_ giant magnetocaloric material. The present direct measurements confirm the exceptionally large cyclic entropy change achievable at an intermediate field (1 or 2 T) in the MnFe(P,Si,B) system.

## Declaration of competing interest

The authors declare that they have no conflicts of interest in this work.

## Appendix Supplementary materials

Image, application 1
